# Toxic Effects of Cadmium Exposure on Hematological and Plasma Biochemical Parameters in Fish: A Review

**DOI:** 10.3390/toxics12100699

**Published:** 2024-09-26

**Authors:** Young-Bin Yu, Ju-Wook Lee, A-Hyun Jo, Young Jae Choi, Cheol Young Choi, Ju-Chan Kang, Jun-Hwan Kim

**Affiliations:** 1 Department of Aquatic Life Medicine, Pukyong National University, Busan 48513, Republic of Korea; unero@naver.com; 2 Incheon Regional Office of National Fishery Products Quality Management Service, Incheon 22346, Republic of Korea; leejuwook84@gmail.com; 3 Department of Aquatic Life Medicine, Jeju National University, Jeju 63243, Republic of Korea; joahyun0902@naver.com; 4 Department of Marine Life Science, Jeju National University, Jeju 63243, Republic of Korea; 5 Inland Fisheries Research Institute, National Institute of Fisheries Science, Geumsan 312844, Republic of Korea; discus62@korea.kr; 6 Division of Marine BioScience, National Korea Maritime and Ocean University, Busan 49112, Republic of Korea

**Keywords:** cadmium toxicity, hematological parameters, plasma biochemical parameters

## Abstract

Cadmium (Cd) is a non-essential trace element that poses significant toxic effects on fish. This review focuses on hematological and plasma biochemical parameters as key indicators of fish health under Cd exposure. Hematological parameters, such as red blood cell (RBC) count, hemoglobin (Hb) concentration, and hematocrit (Ht), were selected for their critical role in oxygen transport and their sensitivity to Cd-induced disruptions, which often result in anemia and impaired oxygen delivery to tissues. Mean corpuscular volume (MCV), mean corpuscular hemoglobin (MCH), and mean corpuscular hemoglobin concentration (MCHC) provide further insights into erythropoiesis and hemoglobin synthesis, both of which are essential for assessing Cd toxicity. Plasma biochemical parameters, including calcium, magnesium, glucose, cholesterol, total protein, and liver enzymes such as aspartate aminotransferase (AST), alanine aminotransferase (ALT), and alkaline phosphatase (ALP), are crucial for understanding ionic balance, metabolic regulation, and organ function, especially in fish exposed to Cd. These biomarkers offer a comprehensive view of the physiological stress and organ damage caused by Cd toxicity. This review synthesizes literature findings on the toxic effects of Cd on these parameters. It also discusses potential mitigation strategies, including dietary supplementation with antioxidants and trace elements, to counteract the harmful effects of Cd exposure.

## 1. Introduction

**Figure 1:**
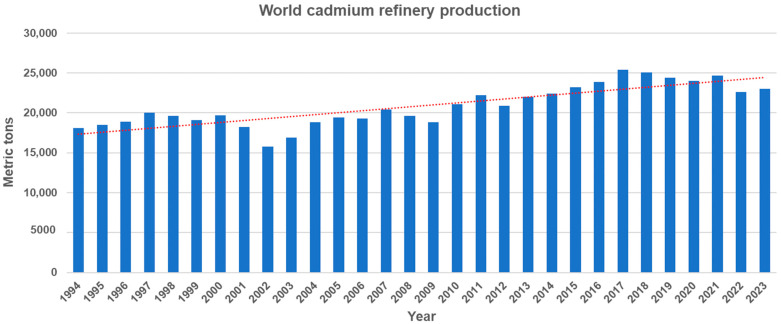
World cadmium refinery production from 1994 to 2023 (Source: U.S Geological Survey, Cadmium statistics and information. In: Mineral Commodity Summaries). The dotted line represents the trend line of cadmium refinery production.

Cadmium (Cd) is a non-essential trace element that is naturally present throughout the environment. It primarily exists in association with zinc sulfide and is found in the Earth’s crust at a concentration of 0.15 mg/kg and in the sea at 1.1 × 10^4^ mg/L [1]. Cd can be released into aquatic environments through natural processes such as volcanic activity, weathering of Cd-containing rocks, and forest fires [2]. Moreover, anthropogenic sources such as mining, smelting, industrial processes, urban runoff, and agricultural activities, significantly increase the Cd concentration in aquatic environments. They contribute approximately 90 percent of the Cd found in these environments, posing significant environmental risks [3]. World Cd production has shown a steady increase from 18,100 tons in 1994 to 23,000 tons in 2023, despite a slight decline in the past seven years (Figure 1). In the U.S., industrial and manufacturing facilities, along with mining operations, disclose the quantities of Cd and Cd compounds released into the environment through the EPA Toxics Release Inventory (TRI) Program. Data from the TRI reveals that the annual average environmental release of Cd and Cd compounds across all industries reached approximately 4.4 million pounds in 2022.

The U.S. EPA recommended national ambient water quality criteria for Cd in 2016 are as follows: In freshwater, the acute criterion specifies that the one-hour maximum concentration should not exceed 0.0018 mg/L at a total hardness of 100 mg/L as CaCO_3_, while the chronic criterion sets a four-day average concentration limit of 0.00072 mg/L under the same hardness conditions. For estuarine and marine environments, the acute criterion allows for a one-hour maximum concentration of up to 0.033 mg/L, and the chronic criterion limits the four-day average concentration to 0.0079 mg/L [3]. Cd generally occurs at low concentrations in the water column, with total dissolved Cd levels below 0.0005 mg/L in freshwater and approximately 0.00002 mg/L in seawater [4]. While Cd concentrations in natural waters are generally low, significantly higher levels can be found in surface waters within Cd-polluted environments. For example, Cd concentrations in the water of the Kali River in India ranged from 0.06 to 0.08 mg/L, indicating substantial contamination in the river [5].

Once introduced into the aquatic environment, Cd interacts with particulate matter, iron oxides, or clay minerals, leading to its accumulation in sediments. However, when continuous inputs of Cd result in high concentrations within the sediment, an imbalance between the concentrations in the water column and the sediment can occur, potentially leading to the remobilization of Cd from the sediment back into the water column [4]. The Cd concentration in sediments can vary widely depending on the location and the extent of contamination. For instance, in the Nile River in Egypt, the concentration of Cd in sediments ranged from 0.09 to 0.38 mg/kg, with an average of 0.16 mg/kg [6]. In contrast, higher concentrations were observed in the sediments of the Yamuna River in India, where Cd levels ranged from 0.82 to 4.6 mg/kg [7]. Similarly, in the Gomti River, also in India, sedimentary Cd concentrations were found to be as high as 0.7 to 7.9 mg/kg, indicating significant contamination [5].

In the aquatic environments, Cd compounds can transition between various phases through processes such as chelation, adsorption, desorption, and precipitation and dissolution [1]. These processes facilitate the exchange of Cd between water and sediment. Cd can form various chemical species including ions and complexes with inorganic ligands (e.g., Cl^−^, SO_4_^2−^, HCO_3_^−^, and F^−^), as well as organic ligands (e.g., amino acids, citrate, oxalate, salicylate, fulvic acid, and humic acid) [8,9]. These complexes influence the solubility and mobility of Cd compounds in water. The various forms of Cd, including both charged and neutrally-charged complexes, influence its bioavailability to aquatic organisms [1]. Charged species generally require specific transport mechanisms to pass across cell membranes. In contrast, the behavior of neutrally charged complexes is chemically similar to non-ionic organic compounds, allowing them to pass across cell membranes freely via passive diffusion [1,10]. Due to these properties of Cd, it is absorbed by aquatic organisms through the gills and epithelial cells of the digestive tract. After uptake, Cd may bind with transport proteins in the plasma and be distributed to various organs via the circulatory system. It forms complexes with small peptides or proteins that contain sulfhydryl groups, such as glutathione, or with metal-binding proteins such as metallothionein. These complexes often attach to RBC surfaces, facilitating their transport throughout body. Additionally, Cd can accumulate in cells by replacing essential divalent cations in calcium channels or by interacting with zinc transporters [11]. Cd toxicity can be further exacerbated by its non-biodegradability and potential for accumulation in tissues, enabling it to exhibit high toxicity even at low concentrations [12]. Cd toxicity can lead to various physiological damage including growth retardation, reduced survival rates, metabolic disturbances, enzyme inhibition, decreased immunity, oxidative stress, and neurological disorder [8,13,14,15,16]. In brief, when fish are exposed to Cd, the energy required to detoxify accumulated Cd in tissues may reduce the energy available for growth, resulting in growth retardation and reduced survival rates [17,18]. Cd exposure increases the nuclear and cytoplasmic deposition of lipid droplets in hepatocyte, leading to disruption of lipid metabolism [19]. Cd produces reactive oxygen species (ROS), leading to significant cellular damage, triggering inflammation, and promoting apoptosis through DNA damage and protein oxidation [12]. Cd exposure causes a significant neurotoxicity to fish by inhibiting sulfhydryl-containing enzyme activity and causing neuronal damage in the brain [20].

Hematological and plasma biochemical parameters serve as crucial biomarkers for assessing health and environmental impacts in fish, providing a sensitive and rapid method to detect physiological changes caused by a variety of stressors, such as toxic pollutants [21,22]. Hematological parameters such as red blood cell (RBC), hemoglobin (Hb), and hematocrit (Ht) were chosen due to their direct involvement in oxygen transport and their sensitivity to changes in RBC production and destruction, which are commonly observed under heavy metal exposure such as Cd. Mean corpuscular volume (MCV), mean corpuscular hemoglobin (MCH), and mean corpuscular hemoglobin concentration (MCHC) provide detailed information on the size and hemoglobin content of red blood cells, allowing for a deeper understanding of erythrocyte health and the potential development of anemia or other hematological disturbances under toxic stress. Calcium and magnesium were selected as they are key electrolytes involved in osmoregulation and ion homeostasis, which are often disrupted by Cd toxicity. Glucose serves as a critical marker of metabolic stress, while cholesterol and total protein levels offer insights into lipid metabolism and nutritional status, which can be affected by environmental pollutants. Enzymes such as alanine aminotransferase (AST), alanine aminotransferase (ALT), and alkaline phosphatase (ALP) were included because they are well-known indicators of liver function and cellular damage, making them particularly relevant for detecting Cd-induced hepatotoxicity. The hematopoietic system of fish reacts sensitively to various environmental toxic substances present in the water, directly affecting hematological and plasma biochemical parameters [23]. Therefore, the analysis of these parameters reflects the extensive physiological changes in fish due to environmental toxic substances, providing comprehensive insights into their adaptive and destructive responses [24].

Hematological and plasma biochemical analysis is relatively non-invasive, rapid, and cost-effective, allowing for the early detection of physiological disturbances before the appearance of external symptoms, enabling the effective management of health status [21,25,26]. However, these parameters are influenced by internal factors including nutritional status, age, size, gender, sexual maturity, as well as external factors including temperature, water quality, toxic substances, diseases, and parasitism [27]. Understanding and interpreting these factors is essential for enhancing the accuracy and reliability of hematological analysis.

In this review, the selection of hematological parameters (RBC, Hb, Ht, MCV, MCH, and MCHC) and plasma biochemical parameters (calcium, magnesium, glucose, cholesterol, total protein, AST, ALT, and ALP) is based on their well-documented sensitivity and relevance in toxicological studies, particularly for evaluating the toxic effects of Cd on fish physiology. These parameters were chosen for their ability to provide a comprehensive yet focused overview of the physiological and biochemical disruptions caused by Cd exposure. While additional parameters could have been included, this review focuses on those with the most established relevance and sensitivity to ensure both clarity and feasibility. It is important to note that the Cd concentrations used in many toxicological studies reviewed in this manuscript often exceed the levels typically found in natural aquatic environments. For instance, experimental Cd exposure concentrations can range from 0.0001 to 24 mg/L, whereas Cd concentrations in natural waters are generally below 0.0005 mg/L in freshwater and even lower in marine environments [4]. These elevated concentrations are not environmentally representative but are employed to investigate the toxic effects of Cd under controlled experimental conditions. Such elevated exposure concentrations are necessary to induce observable toxic effects within controlled exposure periods, enabling a focused study of the physiological and biochemical impacts of Cd on aquatic organisms. Although these exposure concentrations do not reflect typical environmental levels, they are crucial for determining toxic thresholds and understanding the mechanisms of Cd toxicity. Thus, this review aims to provide comprehensive information on the toxic effects of Cd exposure on hematological and plasma biochemical parameters in fish. It synthesizes the literature about the toxic effects of Cd on hematological parameters such as RBC count, Hb, Ht, MCV, MCH, and MCHC. Additionally, it examines alterations in plasma biochemical parameters including calcium and magnesium ions, glucose, cholesterol, total protein, AST, ALT, and ALP.

## 2. Methods

The literature for this review was searched using Google Scholar, with terms such as “cadmium toxicity”, “cadmium hematological parameters”, or “cadmium plasma biochemical parameters” in combination with “fish”. We excluded publications older than 15 years, and selected a total of 21 relevant studies published between 2009 and 2022. The selected studies were reviewed individually and categorized based on fish species, Cd concentration, exposure time, response concentration, and response. The findings are presented in Table 1 and Table 2 for hematological parameters, and in Table 3, Table 4, Table 5, Table 6 and Table 7 for plasma biochemical parameters.

## 3. Hematological Parameters

Cd exposure in fish causes extensive hemorrhage and thrombosis in the heart, liver and kidneys, which directly affects the cardiovascular system with persistent pathological symptoms such as ischemic damage and necrosis in target tissues [28]. Cd exposure in fish leads to the accumulation of Cd in major tissues such as liver, kidney and spleen. This accumulation inhibits functions in these hematopoietic tissues, which can result in anemia by inducing angiogenesis disorders and blood cell destruction [29]. Kondera and Witeska [30] suggest that Cd exposure could interfere with the hematopoietic process by damaging the hematopoietic tissue and destroying all hematopoietic progenitor cells in the head kidney tissue.

### 3.1. RBC Counts, Hb and Ht

RBC counts, Hb and Ht in fish exposed to Cd are demonstrated in Table 1. RBCs are the most abundant cell type with the main function of carrying oxygen to each tissue in fish. They can also serve as a “red blood cell immune system”, functioning in the immune defense like white blood cells [31]. RBCs in fish are produced in hematopoietic tissues such as the kidneys and spleen, and Cd-induced stress can lead to macrocytic anemia by destroying mature RBCs or induce anemia status in fish by inhibiting the production of RBCs and angiogenesis [22]. Fish in various stressful situations tend to have a decrease in RBCs, Ht level and Hb concentration, and the anemia induced by the stress is used as an important indicator to evaluate environmental stress [32]. Ht is a simple measure of the content of RBCs in blood volume as a percentage, and it is an important indicator to evaluate the blood oxygen-carrying capacity of fish [24]. A reduction in Ht is closely related to fish fin bleeding, anemia, and hemodilution caused by various metal exposures or environmental stressors [33]. Hb is an iron-containing oxygen transport metalloprotein in RBCs that functions as an oxygen transport along with an antioxidant and iron metabolism modulator [31].

Abdel-Tawwab and Wafeek [22] reported a significant decrease in RBCs, Ht and Hb in Nile tilapia, *Oreochromis niloticus,* exposed to 0.5 mg/L Cd, which was due to either internal bleeding from Cd stress-induced renal injury or the competitive binding of iron (Fe) for Hb synthesis. Kaoud et al. [34] also reported a significant decrease in RBCs, Ht and Hb in *O. niloticus* exposed to 10 mg/L Cd, which indicates that Cd caused impaired metabolic and hematopoietic activity in fish. Al-Asgah et al. [35] also reported that RBCs, Ht, and Hb in *O. niloticus* were significantly decreased following 3.36 and 5.04 mg/L Cd exposure, which indicates that this was because Cd destroyed mature RBCs, inhibiting erythrocyte production, causing severe anemia. Mekkawy et al. [36] reported a significant decrease in RBCs, Ht and Hb in *O. niloticus* exposed to 4.64 mg/L Cd due to osmotic imbalance in erythrocytes as well as red blood cell destruction and production inhibition. Deen et al. [37] reported a significant decrease in RBCs, Ht and Hb in *O. niloticus* exposed to 10 mg/L Cd, which indicates that haem synthesis was reduced by Cd exposure, leading to mature RBC disruption and production inhibition. Naz et al. [38] also reported a significant decrease in RBCs, Ht and Hb in major carp, *Catla catla,* exposed to 1.35 and 1.8 mg/L Cd. Wang et al. [29] reported that RBC, Ht and Hb of gibel carp, *Carassius auratus gibelio,* were significantly decreased by 1 and 2 mg/L Cd exposure, suggesting that Cd may compete with Fe, causing anemia symptoms due to decreased Hb synthesis. Many authors have reported significant reductions in hematological parameters such as RBCs, Hb and Ht. Various mechanisms of hemotoxicity following Cd exposure include increased mechanical fragility, membrane permeability changes, impaired intestinal uptake and defective Fe metabolism [39,40]. El-Boshy et al. [41] reported a significant decrease in RBCs and Hb in catfish, *Clarias gariepinus*, exposed to 5 and 10 mg/L Cd. Samuel et al. [42] also reported a significant decrease in RBCs and Hb in *C. gariepinus* exposed to 12 mg/L Cd, which indicates that the iron in the blood was reduced, resulting in a decrease in the oxygen transport capacity of the blood, which affected the production of erythrocytes. Pereira et al. [40] reported that RBCs and Hb in silver catfish, *Rhamdia quelen*, were significantly decreased following 0.1 mg/L Cd exposure due to RBCs synthesis reduction. Mosbah et al. [28] reported a significant decrease in RBC counts and Ht levels in European bass, *Dicentrarchus labrax*, after initial 0.5 mg/L Cd exposure, which was attributed to hemolytic anemia caused by RBC membrane homeostasis disruption, inhibited Hb synthesis, and renal erythropoietin secretion decrease. Fazio et al. [43] reported a significant decrease in Ht and Hb in giant river-catfish, *Mystus seenghala*, exposed to 17 mg/L Cd because of impaired hematopoietic organs. Lee et al. [44] reported a significant decrease in Ht and Hb in olive flounder, *Paralichthys olivaceus*, exposed to 0.2 and 0.4 mg/L Cd, which indicates that the hematopoietic disorder caused severe anemia. Zhai et al. [45] also reported a significant decrease in Ht and Hb in *O. niloticus* exposed to 1 mg/L Cd. Zaki et al. [46] also reported a significant decrease in Ht in grey mullet, *Mugil cephalus* exposed to 0.25 mg/L Cd. Ibrahim et al. [47] suggested that 0.12 and 0.36 mg/L Cd exposure increased the rate of RBC destruction by inhibiting heme synthesis and RBC production, and the acute anemia might result from RBC membrane destruction due to excessive ROS production. On the other hand, an increase in RBCs and Hb may occur due to Cd exposure, which was argued to be a compensatory response to RBC damage [48]. Ovie and Ikomi [49] reported a significant decrease in RBCs, Ht and Hb in African snakehead, *Parachanna africana* with increasing 1 and 10 mg/L Cd exposure concentrations, attributing this to hemodilution, RBC destruction and restricted RBC synthesis due to dysregulation of osmotic pressure in the fish gill epithelium. Burgos-Aceves et al. [50] also suggested that Cd exposure can lead to severe anemia in fish due to hemodilution, impaired osmotic pressure regulation and internal bleeding from kidney injury.

**Table 1:** RBC counts, hemoglobin, hematocrit in fish exposed to cadmium.

Hematological Parameters		Fish Species	Cd Concentration (mg/L)	Exposure Time (Days or Hour)	Response Concentration (mg/L)	Response	Reference
Red blood cell (RBC) (10^6^/μL)							
Freshwater	Waterborne exposure	*Oreochromis* *niloticus*	0.5	56 d	0.5	-	[22]
			0.12, 0.36	28 d	0.12, 0.36	-	[47]
			10	7 d	10	-	[34]
				25 d	10	-	
			1.68, 3.36, 5.04	10 d	3.36, 5.04	-	[35]
				20 d	3.36, 5.04	-	
			4.64	15 d	4.64	-	[36]
				30 d	4.64	-	
			10	15 d	10	-	[37]
				45 d	10	-	
			1	28 d	-	x	[45]
		*Carassius* *auratus gibelio*	1, 2	14 d	1, 2	-	[29]
				28 d	1, 2	-	
		*Catla catla*	4.5	96 h	-	x	[38]
			0.9, 1.35, 1.8	30 d	0.9, 1.35, 1.8	-	
		*Clarias* *gariepinus*	0.85, 9.35, 10.28, 11.31, 12.44	96 h	-	x	[13]
			2, 5, 10	21 d	5, 10	-	[41]
			12, 16, 20, 24	28 d	12, 16, 20, 24	-	[42]
				56 d	12, 16, 20, 24	-	
				84 d	12	-	
		*Mystus seenghala*	17	112 d	-	x	[43]
		*Parachanna* *africana*	0.1, 1, 10	21 d	1, 10	-	[49]
		*Rhamdia quelen*	0.0001, 0.001, 0.01, 0.1	15 d	0.1	-	[40]
Seawater	Waterborne exposure	*Dicentrarchus labrax*	0.5	48 h	0.5	-	[28]
Hematocrit (Ht) (%)							
Freshwater	Waterborne exposure	*Oreochromis* *niloticus*	0.5	56 d	0.5	-	[22]
			0.12, 0.36	28 d	0.12, 0.36	-	[47]
			10	7 d	10	-	[34]
				25 d	10	-	
			1.68, 3.36, 5.04	10 d	1.68, 3.36, 5.04	-	[35]
				20 d	1.68, 3.36, 5.04	-	
			4.46	15 d	4.64	-	[36]
				30 d	4.64	-	
			10	15 d	10	-	[37]
				45 d	10	-	
			1	28 d	1	-	[45]
		*Carassius* *auratus gibelio*	1, 2	14 d	1, 2	-	[29]
				28 d	1, 2	-	
		*Catla catla*	4.5	96 h	4.5	-	[38]
			0.9, 1.35, 1.8	30 d	0.9, 1.35, 1.8	-	
		*Mystus seenghala*	17	116 d	17	-	[43]
		*Parachanna* *africana*	0.1, 1, 10	21 d	1, 10	-	[49]
		*Rhamdia quelen*	0.0001, 0.001, 0.01, 0.1	15 d	-	x	[40]
Seawater	Waterborne exposure	*Dicentrarchus labrax*	0.5	48 h	0.5	-	[28]
		*Paralichthys* *olivaceus*	0.05, 0.1, 0.2, 0.4	5 d	0.1, 0.2, 0.4	-	[44]
				10 d	0.1, 0.2, 0.4	-	
Hemoglobin (Hb) (g/dL)							
Freshwater	Waterborne exposure	*Oreochromis* *niloticus*	0.5	56 d	0.5	-	[22]
			0.12, 0.36	28 d	0.12, 0.36	-	[47]
			10	7 d	10	-	[34]
				25 d	10	-	
			1.68, 3.36, 5.04	10 d	1.68, 3.36, 5.04	-	[35]
				10 d	1.68, 3.36, 5.04	-	
			4.64	15 d	4.64	-	[36]
				30 d	4.64	-	
			10	15 d	10	-	[37]
				45 d	10	-	
			1	28 d	1	-	[45]
		*Carassius* *auratus gibelio*	1, 2	14 d	1, 2	-	[29]
				28 d	1, 2	-	
		*Catla catla*	4.5	96 h	4.5	-	[38]
			0.9, 1.35, 1.8	30 d	1.35, 1.8	-	
		*Clarias gariepinus*	0.85, 9.35, 10.28, 11.31, 12.44	96 h	-	x	[13]
			2, 5, 10	21 d	5, 10	-	[41]
			12, 16, 20, 24	28 d	12, 16, 20, 24	-	[42]
				56 d	12, 16, 20, 24	-	
				84 d	12	-	
		*Mystus seenghala*	17	112 d	17	-	[43]
		*Parachanna african* *a*	0.1, 1, 10	21 d	1, 10	-	[49]
		*Rhamdia quelen*	0.0001, 0.001, 0.01, 0.1	15 d	0.1	-	[40]
Seawater	Waterborne exposure	*Dicentrarchus labrax*	0.5	48 h	0.5	-	[28]
		*Mugil cephalus*	0.25	7 d	-	x	[46]
				14 d	0.25	-	
				21 d	0.25	-	
		*Paralichthys* *olivaceus*	0.05, 0.1, 0.2, 0.4	5 d	0.2, 0.4	-	[44]
				10 d	0.2, 0.4	-	

-: decrease, x: no effect.

### 3.2. MCV, MCH, and MCHC

MCV, MCH, and MCHC in fish exposed to Cd are demonstrated in Table 2. MCV and MCH are important indicators for evaluating the diagnosis of anemia in most vertebrates, including fish, concerning the reduction of RBCs and Hb. MCHC is a good indicator for evaluating RBC swelling and reductions in hemoglobin synthesis [36]. These parameters offer crucial insights into fish health, particularly in relation to toxicant-induced changes in oxygen transport capacity and erythropoiesis [24]. The calculations for these parameters are based on standard formulas: MCV = [Ht/RBC counts] × 10, MCH = [Hb/RBC counts] × 10, MCHC = [Hb/Ht] × 100. MCV measures the average volume of individual RBCs and can indicate changes in RBC size caused by environmental stressors, including heavy metals. An increase in MCV may serve as a compensatory mechanism to enhance oxygen transport when fish are under stress induced by toxicants, whereas decreases often reflect RBC damage or impaired erythropoiesis [26]. Cd exposure not only induces oxidative stress but also disrupts iron metabolism, which is essential for Hb synthesis. This disruption can result in changes in MCV due to increased RBC destruction or impaired erythropoiesis. Moreover, hemolysis caused by Cd-induced oxidative stress can reduce MCH and MCHC by decreasing the Hb content in RBCs. MCH quantifies the average amount of Hb per RBC, and MCHC is the average concentration of Hb per volume of RBCs. Alterations in MCH and MCHC values due to Cd exposure can indicate dysfunctional erythropoiesis and impaired Hb synthesis, both of which are critical for oxygen transport and cellular function. As demonstrated by Guo et al. [51], Cd exposure perturbs systemic iron homeostasis, leading to iron overload in fish livers and spleens, and is linked to increased serum iron and transferrin saturation. These disruptions in iron metabolism are often accompanied by hemolysis, where erythrocyte fragility increases, further diminishing the hemoglobin concentration in RBCs. An increase in these values may serve as a compensatory mechanism to enhance oxygen transport when fish are under stress induced by toxicants, whereas decreases often reflect RBC damage or impaired erythropoiesis [26]. Ten mg/L Cd exposure caused significant reductions in MCH and MCV in *P. africana*, indicating RBC shrinkage due to microcytic or hypoxic anemia [49]. Similarly, Mekkawy et al. [36] reported a significant decrease in MCV, MCH and MCHC in *O. niloticus* by 4.64 mg/L Cd exposure. These changes are considered to be caused by impaired hemoglobin synthesis, structural damage to the RBC membrane, and hemolysis. Deen et al. [37] reported a significant increase in MCV, whereas a decrease in MCH and MCHC in *O. niloticus* exposed to 10 mg/L Cd, which indicates that the metabolic activity and hematopoietic function of fish were impaired. Ibrahim et al. [47] reported that MCV and MCH were significantly increased following 0.12 and 0.36 mg/L Cd, whereas MCHC was significantly decreased following 0.36 mg/L Cd in *O. niloticus* due to hemolysis of RBCs and a reduction of iron in Hb. Faizo et al. [43] reported a significant increase in MCV and MCH, whereas a decrease in MCHC in *M*. *seenghala* exposed to 17 mg/L Cd due to hematopoietic organ dysfunction. Ovie and Ikomi [49] reported a significant decrease in MCV and MCH in *P. africana* with 10 mg/L Cd exposure concentrations, attributing this to the reduction of red blood cells due to microcellular anemia. Zhai et al. [45] also reported a significant decrease in MCV in *O. niloticus* exposed to 1 mg/L Cd.

**Table 2:** MCV, MCH, and MCHC in fish exposed to cadmium.

Hematological Parameters		Fish Species	Cd Concentration (mg/L)	Exposure Time (Days or Hour)	Response Concentration (mg/L)	Response	Reference
Mean corpuscular volume (MCV) (fL)							
Freshwater	Waterborne exposure	*Oreochromis niloticus*	4.64	15 d	4.64	-	[36]
				30 d	4.64	-	
			10	15 d	10	+	[37]
				45 d	10	+	
			0.12, 0.36	28 d	0.12, 0.36	+	[47]
			1	28 d	1	-	[45]
		*Clarias gariepinus*	2, 5, 10	21 d	-	x	[41]
			12, 16, 20, 24	28 d	-	x	[42]
				56 d	12	+	
					20, 24	-	
				84 d	12	-	
		*Mystus seenghala*	17	112 d	17	+	[43]
		*Parachanna african* *a*	0.1, 1, 10	21 d	10	-	[49]
		*Rhamdia quelen*	0.0001, 0.001, 0.01, 0.1	15 d	-	x	[40]
Seawater	Waterborne exposure	*Dicentrarchus labrax*	0.5	48 h	-	x	[28]
Mean corpuscular hemoglobin (MCH) (pg)							
Freshwater	Waterborne exposure	*Oreochromis niloticus*	4.64	15 d	4.64	-	[36]
				30 d	4.64	-	
			10	15 d	10	-	[37]
				45 d	10	-	
			0.12, 0.36	28 d	0.12, 0.36	+	[47]
			1	28 d	-	x	[45]
		*Clarias gariepinus*	2, 5, 10	21 d	-	x	[41]
			12, 16, 20, 24	28 d	16	+	[42]
				56 d	12	+	
					24	-	
				84 d	12	-	
		*Mystus seenghala*	17	112 d	17	+	[43]
		*Parachanna african* *a*	0.1, 1, 10	21 d	10	-	[49]
		*Rhamdia quelen*	0.0001, 0.001, 0.01, 0.1	15 d	-	x	[40]
Mean corpuscular hemoglobin concentration (MCHC) (g/dL)							
Freshwater	Waterborne exposure	*Oreochromis niloticus*	4.64	15 d	-	x	[36]
				30 d	4.64	-	
			10	15 d	10	-	[37]
				45 d	10	-	
			0.12, 0.36	28 d	0.36	-	[47]
			1	28 d	-	x	[45]
		*Clarias* *gariepinus*	2, 5, 10	21 d	-	x	[41]
			12, 16, 20, 24	28 d	16, 20, 24	-	[42]
				56 d	12, 16, 20, 24	-	
				84 d	12	-	
		*Mystus seenghala*	17	112 d	17	-	[43]
		*Parachanna* *africana*	0.1, 1, 10	21 d	-	x	[49]
		*Rhamdia quelen*	0.0001, 0.001, 0.01, 0.1	15 d	-	x	[40]
Seawater	Waterborne exposure	*Dicentrarchus labrax*	0.5	48 h	-	x	[28]

+: increase, -: decrease, x: no effect.

## 4. Plasma Biochemical Parameters

### 4.1. Calcium and Magnesium

Calcium and magnesium in fish exposed to Cd are demonstrated in Table 3. Intracellular calcium ions are key regulators of cell survival and play important roles in various cellular processes such as muscle contraction, secretion, metabolism, cell differentiation and apoptosis as well as various signaling pathways in cells [52]. Magnesium ions play a major role as cofactors for many enzymes and are essential elements of proteins and nucleic acids, acting as a modulator of ion channels. Moreover, magnesium is necessary for the binding of substances to the plasma membrane, as well as for mitochondrial and cytoskeletal integrity and nucleic acid and protein synthesis [53]. The inorganic components such as calcium and magnesium are important indicators for evaluating the toxic effect on ion homeostasis in fish exposed to metals [54]. Cd interferes with the homeostasis of calcium and magnesium through its interaction with ion channels and ATPases. Due to its physicochemical similarity to calcium, Cd can substitute for calcium in various calcium-binding proteins, such as calmodulin, thereby disrupting calcium-dependent signaling pathways. This interference with calcium signaling is a key factor in Cd-induced toxicity. Furthermore, Cd inhibits the activity of calcium/magnesium-ATPase, impairing the regulation of both calcium and magnesium [55]. Lee et al. [44] reported a significant decrease in plasma calcium in *P. olivaceus* following exposure to 0.2 and 0.4 mg/L Cd. This decrease was attributed to calcium malabsorption due to the competitive action of Cd and calcium-ATPase inhibition, leading to hypocalcemia and calcium ion homeostasis impairment. Wang et al. [29] also reported that plasma calcium of *C. gibelio* was significantly decreased by 1 and 2 mg/L Cd exposure. On the contrary, Zaki et al. [46] reported a significant increase in plasma calcium in *M. cephalus* following exposure to 0.25 mg/L Cd, suggesting that this is due to necrosis in the proximal convoluted tubules, which are closely involved in the reabsorption of electrolytes such as calcium in kidney tissue. Wang et al. [29] reported that 1 and 2 mg/L Cd exposure significantly decreased plasma magnesium in *C. auratus gibelio*, which appears to be due to the disturbance of ion homeostasis by Cd exposure. Lee et al. [44] also reported that 0.2 and 0.4 mg/L Cd exposure caused a significant decrease in plasma magnesium in *P. olivaceus*. Cd exposure results in significant changes in plasma calcium and magnesium, leading to disruptions in ionic homeostasis.

**Table 3:** Calcium and magnesium in fish exposed to cadmium.

Plasma Biochemical Parameters		Fish Species	Cd Concentration (mg/L)	Exposure Time (Days or Hour)	Response Concentration (mg/L)	Response	Reference
Ca (mg/dL)							
Freshwater	Waterborne exposure	*Carassius* *auratus gibelio*	1, 2	14 d	1, 2	-	[29]
				28 d	1, 2	-	
Seawater	Waterborne exposure	*Mugil cephalus*	0.25	7 d	-	x	[46]
				14 d	0.25	+	
				21 d	0.25	+	
		*Paralichthys* *olivaceus*	0.05, 0.1, 0.2, 0.4	5 d	0.2, 0.4	-	[44]
				10 d	0.2, 0.4	-	
Mg (mg/dL)							
Freshwater	Waterborne exposure	*Carassius* *auratus gibelio*	1, 2	14 d	1, 2	-	[29]
				28 d	1, 2	-	
Seawater	Waterborne exposure	*Paralichthys* *olivaceus*	0.05, 0.1, 0.2, 0.4	5 d	0.4	-	[44]
				10 d	0.2, 0.4	-	

+: increase, -: decrease, x: no effect.

### 4.2. Glucose

Glucose in fish exposed to Cd is demonstrated in Table 4. Plasma glucose is a major indicator of fish stress induced by metal exposure, and elevated glucose levels reflect stress status in carbohydrate metabolism. Generally, carbohydrate metabolism in fish under environmental stress is increased, and nephrotoxicity and glucose homeostasis are disturbed, leading to hyperglycemia [56]. Lee et al. [44] suggested that Cd exposure induces inhibition of energy metabolism in fish, stimulation of gluconeogenesis enzymes, an increase in lipid peroxidation, excessive oxidative damage, DNA and membrane structure changes, insulin receptor reduction through thiol protein changes, and metalloenzyme interference. Cd-induced hyperglycemia can lead to impaired glucose homeostasis and metabolic control. Ibrahim et al. [47] reported that plasma glucose was significantly increased following 0.12 and 0.36 mg/L Cd, suggesting that this was because glycolysis was promoted to provide energy to counter Cd toxicity. Tabat et al. [57] reported a significant increase in plasma glucose in *C. gariepinus* fingerlings exposed to 0.41, 0.81 and 1.62 mg/L Cd and *C. gariepinus* juvenile exposed to 2.03 mg/L Cd, which indicates that long-term exposure of Cd caused chronic stress, converting amino acids and glycerol present in the blood into glucose. El-Boshy et al. [41] reported a significant increase in plasma glucose in *C. gariepinus* exposed to 2, 5 and 10 mg/L Cd, which indicated that hyperglycemia was induced by increased glycogen. Miandare et al. [58] reported that 0.2 and 0.8 mg/L Cd exposure induced an increase in plasma glucose levels in Persian sturgeon, *Acipenser persicus*, which is judged to be a stress response following Cd exposure. Heydarnejad et al. [59] suggested that 0.001 and 0.003 mg/L Cd exposure induced an increase in plasma glucose levels in rainbow trout, *Oncorhynchus mykiss*, by glycogenolysis, and the changes in the plasma glucose were associated with kidney and liver damage. Similarly, Fazio et al. [43] reported that 17 mg/L Cd exposure significantly increased plasma glucose levels in *M. seenghala*, attributing the results to the conversion of stored glycogen in muscles and liver to meet energy demands to counteract Cd toxicity. Mekkawy et al. [36] reported that 4.64 mg/L Cd exposure significantly increased plasma glucose levels in *O. niloticus*, and the hyperglycemia might be due to the activation of the hepatic gluconeogenesis or the stimulation of plasma catecholamines and corticosteroid hormones. Al-Asgah et al. [35] also reported that 1.68, 3.36, and 5.04 mg/L Cd exposure induces a decrease in plasma glucose in *O. niloticus*. Wang et al. [29] also reported that the plasma glucose of *C*. *auratus gibelio* was significantly increased by 1 and 2 mg/L Cd exposure. Zaki et al. [46] reported a significant increase in plasma glucose in *M. cephalus* exposed to 0.25 mg/L Cd, which indicates that Cd exposure caused stress, resulting in a rapid secretion of glucocorticoids and catecholamines from the adrenal glands, leading to hyperglycemia. Lee et al. [44] reported a significant increase in plasma glucose in *P. olivaceus* following exposure to 0.4 mg/L Cd, which suggests that glucose homeostasis and metabolic regulation disorders have affected the physiological state of fish. On the contrary, Ovie and Ikomi [49] reported a significant decrease in plasma glucose in *P. africana* with increasing 0.1, 1 and 10 mg/L Cd exposure concentrations.

**Table 4:** Glucose in fish exposed to cadmium.

Plasma Biochemical Parameters		Fish Species	Cd Concentration (mg/L)	Exposure Time (Days or Hour)	Response Concentration (mg/L)	Response	Reference
Glucose (mg/dL)							
Freshwater	Waterborne exposure	*Oreochromis* *niloticus*	0.12, 0.36	28 d	0.12, 0.36	+	[47]
			1.68, 3.36, 5.04	10 d	1.68, 3.36, 5.04	+	[35]
				20 d	1.68, 3.36, 5.04	+	
			4.64	15 d	4.64	+	[36]
				30 d	4.64	+	
		*Oncorhynchus m* *y* *kiss*	0.001, 0.003	1 d	-	x	[59]
				15 d	0.001, 0.003	+	
				30 d	0.001	+	
		*Carassius* *auratus gibelio*	1, 2	14 d	1, 2	+	[29]
				28 d	1, 2	+	
		*Clarias* *gariepinus* fingerlings	0.41, 0.81, 1.62	56 d	0.41, 0.81, 1.62	+	[57]
		*Clarias* *gariepinus* juvenile	0.51, 1.02, 2.03	56 d	2.03	+	
		*Clarias* *gariepinus*	2, 5, 10	21 d	2, 5, 10	+	[41]
		*Mystus seenghala*	17	112 d	17	+	[43]
		*Parachanna* *africana*	0.1, 1, 10	21 d	0.1, 1, 10	-	[49]
Seawater	Waterborne exposure	*Acipenser* *persicus*	0.2, 0.8	1 d	0.8	+	[58]
				2 d	0.8	+	
				4 d	0.2, 0.8	+	
				7 d	0.2, 0.8	+	
				14 d	-	x	
		*Mugil cephalus*	0.25	7 d	-	x	[46]
				14 d	0.25	+	
				21 d	0.25	+	
		*Paralichthys* *olivaceus*	0.05, 0.1, 0.2, 0.4	5 d	0.4	+	[44]
				10 d	0.2, 0.4	+	

+: increase, -: decrease, x: no effect.

### 4.3. Cholesterol

Cholesterol in fish exposed to Cd is demonstrated in Table 5. Cholesterol is an essential component for maintaining cell morphology in lipid bilayer cell membranes, as well as for cell recognition or signaling pathways. It is involved in endocrine system regulation and physiological regulation as a precursor of various steroid hormones such as corticosteroid [60]. Metal exposure in fish causes trans-membrane gradient alterations, stimulation or inhibition of lipid metabolism, hormone changes associated with lipid metabolism, and cell membrane damage. Therefore, a significant change in plasma cholesterol levels is used as a major indicator of metal exposure stress [61,62]. Tabat et al. [58] reported that 2.03 mg/L Cd exposure significantly increased plasma cholesterol in *C. garipinus*, suggesting that the results were due to higher energy requirements under Cd-induced toxic stress. Lee et al. [44] reported that 0.4 mg/L Cd exposure significantly increased plasma cholesterol in *P. olivaceus*, attributing the increase to the termination of the biosynthetic metabolism of lipids and lipoproteins in the liver by Cd exposure. Zhai et al. [45] reported that 1 mg/L Cd exposure induced significant changes in plasma cholesterol in *O. niloticus*, suggesting that Cd exposure adversely affects lipid metabolism. Heydarnejad et al. [59] reported a significant decrease in plasma cholesterol in exposed to 0.003 mg/L Cd for 1 and 15 days, whereas an increase in plasma cholesterol exposed to 0.003 mg/L Cd for 30 days in *O. mykiss*. This significant alteration in plasma cholesterol indicates that Cd affected the cell membrane, causing stress.

**Table 5:** Cholesterol in fish exposed to cadmium.

Plasma Biochemical Parameters		Fish Species	Cd Concentration (mg/L)	Exposure Time (Days or Hour)	Response Concentration (mg/L)	Response	Reference
Cholesterol (mg/dL)							
Freshwater	Waterborne exposure	*Oncorhynchus m* *y* *kiss*	0.001, 0.003	1 d	0.001, 0.003	-	[59]
				15 d	0.003	-	
				30 d	0.003	+	
		*Oreochromis* *niloticus*	1	28 d	1	-	[45]
		*Clarias* *gariepinus* fingerlings	0.41, 0.81, 1.62	56 d	-	x	[57]
		*Clarias* *gariepinus* juvenile	0.51, 1.02, 2.03	56 d	2.03	+	
Seawater	Waterborne exposure	*Paralichthys* *olivaceus*	0.05, 0.1, 0.2, 0.4	5 d	0.4	+	[44]

+: increase, -: decrease, x: no effect.

### 4.4. Total Protein

Total protein in fish exposed to Cd is demonstrated in Table 6. Plasma proteins play an important role in osmotic balance and blood circulation, and protein content is an important indicator for understanding the physiological mechanisms underlying health status alterations and various environmental stresses [63]. Plasma protein content can be reduced by impaired food intake as well as increased homeostatic energy costs, including recovery from tissue damage and detoxification from toxic exposure [64]. Ibrahim et al. [47] reported that 0.12 and 0.36 mg/L Cd exposure significantly decreased plasma total protein in *O. niloticus*, suggesting that Cd toxicity may have a direct effect on carbonyl protein production through oxidation, leading to hepatocellular degeneration due to decreased protein biosynthesis. Al-Asgah et al. [35] reported that 1.68, 3.36, and 5.04 mg/L Cd exposure induces a decrease in total protein in *O. niloticus*, which was due to protein synthesis disruption in subcellular structures and hepatic synthesis inhibition in blood proteins. Mekkawy et al. [36] reported that 4.64 mg/L Cd exposure significantly decreased plasma total protein levels in *O. niloticus* due to the structural destruction of protein synthesis cells and inhibition of hepatic synthesis. Zhai et al. [45] reported that 1 mg/L Cd exposure significantly decreased plasma total protein in *O. niloticus*, attributing it to the adverse effects of Cd exposure on protein metabolism. El-Boshy et al. [41] reported a significant decrease in plasma total protein in *C. gariepinus* exposed to 10 mg/L Cd due to liver and kidney damage. Faizo et al. [43] reported a significant decrease in plasma total protein in *M. seenghala* exposed to 17 mg/L Cd. Zaki et al. [46] reported a significant decrease in plasma total protein in *M. cephalus* exposed to 0.25 mg/L Cd. Ovie and Ikomi [49] also reported a significant decrease in plasma total protein in *P. africana* with increasing 1 and 10 mg/L Cd exposure concentrations. On the other hand, Heydarnejad et al. [59] reported a significant increase in the plasma total protein in *O. mykiss*, which indicated that the significant alteration in total protein was due to liver damage and protein loss. Wang et al. [29] also reported a significant increase in plasma total protein of *C. auratus gibelio* with 1 and 2 mg/L Cd exposure, which is due to plasma release from Cd-induced tissue damage and dysfunction. Tabat et al. [57] argued that 2.03 mg/L Cd exposure caused a significant increase in plasma total protein in *C. garipinus* due to the loss of plasma water induced by Cd exposure. Cd exposure causes an accumulation of liver tissue and liver injury due to impaired osmotic pressure regulation, tissue necrosis, hemodilution and an increased energy demand for toxic detoxification. In addition, Cd accumulated in liver tissue binds to metallothionein and interferes with cellular mechanisms, thereby increasing the plasma protein concentration.

**Table 6:** Total protein in fish exposed to cadmium.

Plasma Biochemical Parameters		Fish Species	Cd Concentration (mg/L)	Exposure Time (Days or Hour)	Response Concentration (mg/L)	Response	Reference
Total protein (g/dL)							
Freshwater	Waterborne exposure	*Oreochromis* *niloticus*	0.12, 0.36	28 d	0.12, 0.36	-	[47]
			1.68, 3.36, 5.04	10 d	1.68, 3.36, 5.04	-	[35]
				20 d	1.68, 3.36, 5.04	-	
			4.64	15 d	4.64	-	[36]
				30 d	4.64	-	
			1	28 d	1	-	[45]
		*Oncorhynchus m* *y* *kiss*	0.001, 0.003	1 d	0.003	+	[59]
				15 d	0.001, 0.003	+	
				30 d	0.001, 0.003	+	
		*Carassius* *auratus gibelio*	1, 2	14 d	2	-	[29]
				28 d	1, 2	-	
		*Clarias* *gariepinus* fingerlings	0.41, 0.81, 1.62	56 d	-	x	[57]
		*Clarias* *gariepinus* juvenile	0.51, 1.02, 2.03	56 d	2.03	+	
		*Clarias* *gariepinus*	2, 5, 10	21 d	10	-	[41]
		*Mystus seenghala*	17	112 d	17	-	[43]
		*Parachanna* *africana*	0.1, 1, 10	21 d	1, 10	-	[49]
Seawater	Waterborne exposure	*Mugil cephalus*	0.25	7 d	0.25	-	[46]
				14 d	0.25	-	
				21 d	0.25	-	
		*Paralichthys* *olivaceus*	0.05, 0.1, 0.2, 0.4	5 d	-	x	[44]

+: increase, -: decrease, x: no effect.

### 4.5. AST, ALT and ALP

AST, ALT, and ALP in fish exposed to Cd are demonstrated in Table 7. AST is a critical enzyme distributed in RBCs and skeletal muscles in addition to hepatocytes, while ALT is an enzyme that is particularly abundant in hepatocytes. When cells are necrotic or destroyed due to various stressors in fish, AST and ALT are leaked into the bloodstream, serving as a major indicator to evaluate liver damage caused by exposure to various toxicants [65]. Ibrahim et al. [47] reported that 0.12 and 0.36 mg/L Cd exposure increased AST and ALT activities in *O. niloticus*, suggesting that Cd exposure may induce liver cytotoxicity through changes in physiological function and cell membrane stability. Kaoud et al. [34] also reported that 10 mg/L Cd exposure significantly increased AST and ALT activity in *O. niloticus*, which was due to damage or degradation of liver, spleen or muscle tissue. Al-Asgah et al. [35] reported that AST and ALT activity in *O. niloticus* also increased significantly with the 1.68, 3.36 and 5.04 mg/L Cd exposure concentration increased, which is the result of leakage from the liver into the bloodstream due to hepatocellular tissue damage. Mekkawy et al. [36] reported that 4.64 mg/L Cd exposure significantly increased AST and ALT activities in *O. niloticus* and it has been shown that Cd affects liver tissue, causing hepatocellular damage. Zhai et al. [45] reported that 1 mg/L Cd exposure significantly increased AST and ALT activities in *O. niloticus* due to the dysfunction of tissues such as the liver and heart. Wang et al. [29] reported a significant increase in AST and ALT activities in *C. auratus gibelio* with 1 and 2 mg/L Cd exposure, which indicated that plasma enzyme components were released into the plasma due to tissue damage and dysfunction. Heydarnejad et al. [59] reported that 0.001 and 0.003 mg/L Cd exposure significantly increased AST and ALT in *O. mykiss* due to their release into plasma following tissue damage and dysfunction induced by Cd exposure. El-Boshy et al. [41] suggested that 5 and 10 mg/L Cd exposure may result in a significant increase in Kupffer cells, leading to the swelling and rupture of parenchymal cells, focal necrosis, vacuoles filled with cellular debris, and loss of spinal structure. They reported a significant increase in the AST and ALT activities in *C. gariepinus* exposed to Cd. Fazio et al. [43] reported that 17 mg/L Cd exposure significantly increased AST and ALT activities in *M. seenghala* due to the release of enzymes from liver tissue to plasma by liver damage. Lee et al. [44] reported a significant increase in AST and ALT activities in *P. olivaceus* following exposure to 0.4 mg/L Cd, which indicates that Cd exposure caused tissue damage, affecting amino acid and protein metabolic activities. Zaki et al. [46] reported a significant increase in AST and ALT activities in *M. cephalus* exposed to 0.25 mg/L Cd due to liver and kidney damage. Miandare et al. [58] reported that 0.2 and 0.8 mg/L Cd exposure can result in damage to cell membrane and organelle, leading to release from the cytoplasm to the cell membrane. They reported that Cd exposure induced a significant increase in plasma AST and ALT in *A. persicus*.

ALP is a major enzyme in fish metabolism, produced mainly in liver tissue and bone. It plays an important role in transporting metabolites across membranes; ALP generally increases in conditions that affect the liver, including tumors. Javed et al. [66] suggested that ALP is a membrane-bound enzyme found in the bile poles of hepatocytes, and is commonly increased by tissue membrane disruption in pathological conditions such as metal exposure-induced liver damage, bone disease, and renal dysfunction. Ibrahim et al. [47] reported that 0.12 and 0.36 mg/L Cd exposure significantly decreased the ALP activity in *O. niloticus*, which was due to changes in the stability of cell membranes in fish liver by Cd exposure. Wang et al. [29] also reported a significant decrease in ALP in *C. auratus gibelio* with 1 and 2 mg/L Cd exposure. On the contrary, Heydarnejad et al. [59] reported that 0.001 and 0.003 mg/L Cd exposure significantly increased ALP in *O. mykiss*, indicating physiological and functional changes following Cd exposure. Tabat et al. [57] reported that 0.81, 1.62 and 2.03 mg/L Cd exposure significantly increased the ALP in *C. garipinus*, attributing this to cell damage such as apoptosis or necrosis. Lee et al. [44] reported that Cd exposure significantly increased the ALP in *P. olivaceus* due to the induction of hepatic lesion formation such as necrosis, degeneration, and lymphocyte infiltration.

**Table 7:** AST, ALT, and ALP in fish exposed to cadmium.

Plasma Biochemical Parameters		Fish Species	Cd Concentration (mg/L)	Exposure Time (Days or Hour)	Response Concentration (mg/L)	Response	Reference
Alanine aminotransferase (AST) (μ/L)							
Freshwater	Waterborne exposure	*Oreochromis* *niloticus*	0.12, 0.36	28 d	0.12, 0.36	+	[47]
			10	7 d	10	+	[34]
				25 d	10	+	
			1.68, 3.36, 5.04	10 d	1.68, 3.36, 5.04	+	[35]
				20 d	1.68, 3.36, 5.04	+	
			4.64	15 d	4.64	+	[36]
				30 d	4.64	+	
			1	28 d	1	+	[45]
		*Oncorhynchus m* *y* *kiss*	0.001, 0.003	1 d	0.001, 0.003	+	[59]
				15 d	0.001	-	
				30 d	0.001, 0.003	+	
		*Carassius* *auratus gibelio*	1, 2	14 d	1, 2	+	[29]
				28 d	1, 2	+	
		*Clarias* *gariepinus*	2, 5, 10	21 d	5, 10	+	[41]
		*Mystus seenghala*	17	112 d	17	+	[43]
Seawater	Waterborne exposure	*Acipenser* *persicus*	0.2, 0.8	1 d	-	x	[58]
				2 d	0.8	+	
				4 d	0.2, 0.8	+	
				7 d	0.2, 0.8	+	
				14 d	0.8	+	
		*Paralichthys* *olivaceus*	0.05, 0.1, 0.2, 0.4	5 d	0.4	+	[44]
				10 d	0.2, 0.4	+	
		*Mugil cephalus*	0.25	7 d	-	x	[46]
				14 d	0.25	+	
				21 d	0.25	+	
Alanine aminotransferase (ALT) (μ/L)							
Freshwater	Waterborne exposure	*Oreochromis* *niloticus*	0.12, 0.36	28 d	0.12, 0.36	+	[47]
			10	7 d	10	+	[34]
				25 d	10	+	
			1.68, 3.36, 5.04	10 d	1.68, 3.36, 5.04	+	[35]
				20 d	1.68, 3.36, 5.04	+	
			4.64	15 d	4.64	+	[36]
				30 d	4.64	+	
			1	28 d	1	+	[45]
		*Oncorhynchus m* *y* *kiss*	0.001, 0.003	1 d	-	x	[59]
				15 d	0.001, 0.003	+	
				30 d	0.001, 0.003	+	
		*Carassius* *auratus gibelio*	1, 2	14 d	1, 2	+	[29]
				28 d	1, 2	+	
		*Clarias* *gariepinus*	2, 5, 10	21 d	5, 10	+	[41]
		*Mystus seenghala*	17	112 d	17	+	[43]
Seawater	Waterborne exposure	*Acipenser* *persicus*	0.2, 0.8	1 d	0.8	+	[58]
				2 d	0.2, 0.8	+	
				4 d	0.2, 0.8	+	
				7 d	0.2, 0.8	+	
				14 d	0.8	+	
		*Paralichthys* *olivaceus*	0.05, 0.1, 0.2, 0.4	5 d	0.4	+	[44]
				10 d	0.2, 0.4	+	
		*Mugil cephalus*	0.25	7 d	-	x	[46]
				14 d	0.25	+	
				21 d	0.25	+	
Alkaline phosphatase (ALP) (μ/L)							
Freshwater	Waterborne exposure	*Oreochromis* *niloticus*	0.12, 0.36	28 d	0.12, 0.36	-	[47]
		*Oncorhynchus m* *y* *kiss*	0.001, 0.003	1 d	-	x	[59]
				15 d	0.003	+	
				30 d	0.001, 0.003	+	
		*Carassius* *auratus gibelio*	1, 2	14 d	1, 2	-	[29]
				28 d	1, 2	-	
		*Clarias* *gariepinus* fingerlings	0.41, 0.81, 1.62	56 d	0.81, 1.62	+	[57]
		*Clarias* *gariepinus* juvenile	0.51, 1.02, 2.03	56 d	2.03	+	
Seawater	Waterborne exposure	*Paralichthys* *olivaceus*	0.05, 0.1, 0.2, 0.4	5 d	-	x	[44]

+: increase, -: decrease, x: no effect.

## 5. Interactions between Hematological and Plasma Biochemical Parameters

**Figure 2:**
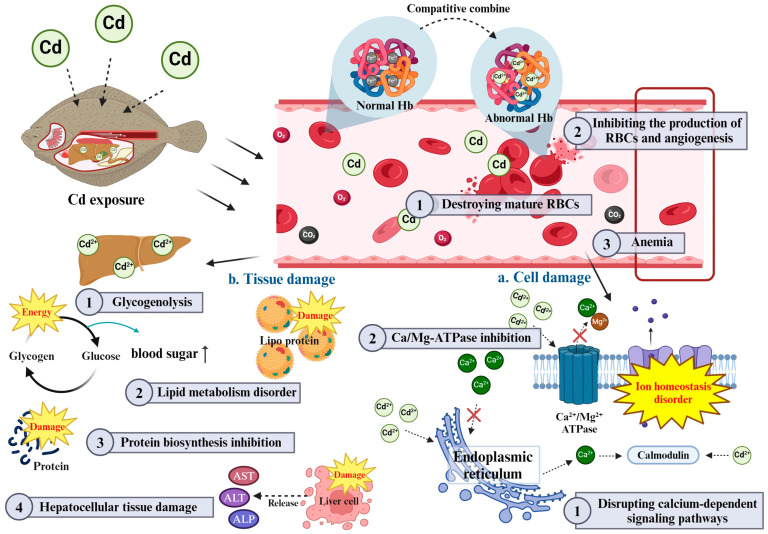
Schematic diagram of Cd toxicity on interaction between hematological and plasma biochemical parameters in fish.

While individual hematological and plasma biochemical parameters offer insight into the physiological impacts of Cd exposure, these parameters do not function in isolation. For instance, the observed decrease in RBC counts Hb and Ht not only suggests impaired oxygen transport but also interacts with changes in plasma protein levels and enzyme activities, highlighting a broader systemic response to Cd toxicity [8]. The reduced oxygen-carrying capacity due to anemia may place additional stress on the liver and kidneys, which is further evidenced by elevated AST, ALT and ALP levels [29]. Additionally, changes in ionic balance (e.g., calcium and magnesium) can cause metabolic imbalances that manifest in glucose and cholesterol levels and ultimately lead to a cascade of toxic effects that can lead to organ damage [67,68]. These interactions underscore a complex, multi-faceted disruption in fish physiology, where hematological and biochemical markers are interconnected (Figure 2).

## 6. Conclusions

This review evaluated the toxicity of Cd exposure on hematological and plasma biochemical parameters in various fish species. The findings indicate that Cd toxicity leads to significant alterations in these parameters, which serve as essential biomarkers for assessing the health status of fish. Cd exposure in fish results in hematological changes such as a decreased RBC count, Hb concentration, and Ht levels, all of which contribute to anemia and impaired oxygen transport. Additionally, alterations in MCV, MCH, and MCHC are observed, indicating various anemic conditions and disturbances in hemoglobin synthesis. Plasma biochemical parameters are also significantly affected by Cd exposure. Notable changes include decreased levels of calcium and magnesium ions, which reflect disrupted ionic homeostasis. Elevated plasma glucose and cholesterol levels indicate metabolic stress and alterations in carbohydrate and lipid metabolism. Furthermore, changes in total protein and enzyme activities (AST, ALT, and ALP) suggest organ damage, particularly in the liver and kidneys, highlighting the toxic effects of Cd on these vital organs. These findings emphasize the importance of hematological and plasma biochemical parameters for assessing the health status of fish in environments contaminated with Cd. These biomarkers are essential for monitoring aquatic ecosystems contaminated by heavy metals and can inform strategies to mitigate the adverse effects of Cd exposure. To alleviate the symptoms of Cd toxicity in fish, several potential approaches can be considered. Dietary supplementation with antioxidants, such as vitamins C and E, may help reduce oxidative stress caused by Cd exposure [8,69]. Additionally, essential trace elements like selenium and zinc could support the fish’s antioxidant defense system, helping to counteract the effects of Cd toxicity [70,71,72,73]. Improving water quality through regular monitoring and reducing sources of Cd contamination will also be crucial in preventing further damage. Effective environmental management, such as maintaining optimal water quality and minimizing stress factors, may strengthen fish resilience against Cd toxicity. Future research should aim to explore the mechanisms of interaction between these hematological and biochemical parameters to better understand how Cd disrupts the overall physiology of fish. Studies investigating the long-term effects of low-dose Cd exposure and its impact on fish populations in natural environments would also be invaluable. Such research could contribute to the development of comprehensive toxicological indicators and inform policies for protecting aquatic ecosystems from heavy metal contamination.

## Data Availability

Not applicable.
